# Proximal Sealing in Zone 1–2 Using the Castor Stent-Graft: Early Results from an Italian Multicenter Registry

**DOI:** 10.3390/medsci14020185

**Published:** 2026-04-07

**Authors:** Antonio Rizza, Simona Sica, Marco Ferraresi, Giovanni Tinelli, Yamume Tshomba, Giovanni Rossi, Giancarlo Trimarchi, Ilenia Foffa, Luca Bastiani, Silvia Di Sibio, Michele Murzi, Cataldo Palmieri, Nicola Tusini, Carmelo Ricci, Andrea Colli, Antonio Lorido, Francesco Talarico, Mafalda Massara, Chang Shu, Sergio Berti, SOCIAL Collaborative Study Group

**Affiliations:** 1 Fondazione Toscana Gabriele Monasterio, 54100 Massa, Italy; giancarlo.trimarchi18@gmail.com (G.T.); ilenia.foffa@cnr.it (I.F.); luca.bastiani@cnr.it (L.B.); disibio@ftgm.it (S.D.S.); michele.murzi@ftgm.it (M.M.); palmieri@ftgm.it (C.P.); berti@ftgm.it (S.B.); 2 Unit of Vascular Surgery, Fondazione Policlinico Universitario A. Gemelli I.R.C.C.S., 00168 Rome, Italygiovanni.tinelli@unicatt.it (G.T.); yamume.tshomba@unicatt.it (Y.T.); 3 Division of Vascular Surgery, Università Cattolica del Sacro Cuore, 00168 Rome, Italy; 4 Vascular Surgery Unit, Manzoni Hospital, 23900 Lecco, Italy; marco.ferraresi7@gmail.com (M.F.); g.rossi@asst-lecco.it (G.R.); 5 Interdisciplinary Center for Health Sciences, Scuola Superiore Sant’Anna, 56127 Pisa, Italy; 6 Institute of Clinical Physiology, National Research Council, 54100 Massa, Italy; 7 Vascular Surgery Unit, Arcispedale S. Maria Nuova, 42123 Reggio Emilia, Italy; nicola.tusini@ausl.re.it; 8 Interventional Radiology Unit, Siena University Hospital, 53100 Siena, Italy; riccar65@gmail.com; 9 Division of Cardiac Surgery, Department of Surgical, Medical and Molecular Pathology and Critical Care, University of Pisa, 56127 Pisa, Italy; colli.andrea.bcn@gmail.com; 10 Unit of Vascular Surgery, Ospedale Belcolle, 01100 Viterbo, Italy; alorido@omceoromapec.it; 11 Vascular Surgery Unit, Ospedale Civico di Palermo, 90127 Palermo, Italy; talarico.vascolare@tiscali.it; 12 Divisione di Chirurgia Vascolare ed Endovascolare, GOM di Reggio Calabria, 89121 Reggio Calabria, Italy; drmafaldamassara@gmail.com; 13 Center of Vascular Surgery, Fuwai Hospital of Chinese Academy of Medical Sciences, Beijing 100037, China; changshu01@fuwaihospital.org

**Keywords:** Castor stent-graft, multicenter registry, TEVAR, outer branch

## Abstract

**Objective:** To evaluate early and mid-term outcomes of thoracic endovascular aortic repair (TEVAR) using the Castor single-branched aortic stent graft in a real-world multicenter Italian experience. **Methods:** This retrospective, nonrandomized, multicenter study included all consecutive patients treated with the Castor stent graft between January 2019 and April 2025 in eight Italian centers. The device was used in patients with thoracic aortic pathologies requiring TEVAR in proximal landing zones 1 or 2. Primary endpoints included technical success, intraoperative major adverse events (MAEs), and deployment accuracy. Secondary endpoints were aortic-related mortality, neurological complications, reinterventions, and endoleaks. **Results:** Fifty-one patients (mean age 68.8 ± 8 years, 75.5% male) were treated, primarily for type B aortic dissection (45.1%) and thoracic aortic aneurysm (27.5%). Proximal landing was in zone 2 in 92.1% and zone 1 in 7.8% of cases. The technical success rate was 94.1%, with three cases (5.8%) of intraoperative type Ia endoleak. No intraoperative deaths or major adverse events occurred. Two cases of minor embolic stroke (3.9%) were observed, both in zone 1 procedures involving left common carotid artery revascularization. At a median follow-up of 22.3 months (range 2–58), no additional endoleaks or neurological events were reported, with 100% branch patency rate. **Conclusions:** The Castor single-branched stent graft is a feasible option for TEVAR in zone 2, with high technical success and low rates of neurological complications. Accurate case selection and procedural planning are essential.

## 1. Introduction

In the last decade, thoracic endovascular aortic repair (TEVAR) has become the first-line treatment for conditions affecting the distal aortic arch and descending thoracic aorta [1]. One of the main technical challenges in these procedures is securing a stable proximal landing zone (PLZ), which often requires the coverage of the left subclavian artery (LSA). In these cases, LSA revascularization is essential to mitigate risks such as posterior stroke, upper limb malperfusion, spinal cord, and myocardial ischemia [2]. LSA revascularization has relied on hybrid approaches combining TEVAR with extra-anatomic bypasses or surgical transpositions [3]. However, the advent of total endovascular approaches has broadened the treatment approaches with different techniques, including branched, scalloped, or fenestrated endografts, chimney, in situ fenestration, and physician-modified endografts [4,5]. Among the latest innovations, the Castor (Microport Medical Co., Ltd., Shanghai, China) single-branched stent graft is a unibody device featuring an integrated outer branch designed specifically for LSA revascularization for aortic dissection [6]. This Italian multicenter study aims to assess early and mid-term outcomes of the Castor stent-graft use in the routine clinical practice for patients with distal aortic arch and descending thoracic aorta pathologies.

## 2. Materials and Methods

### 2.1. Study Design

This was a retrospective, single-arm, nonrandomized, multicenter study involving all consecutive patients treated with Castor single-branched stent grafts (MicroPort Medical, Shanghai, China) across eight Italian centers between 1 January 2019 and 1 April 2025. The study was conducted in accordance with the Declaration of Helsinki and the Institutional Ethics Committee rules. Ethical review and approval were waived due to the retrospective nature of the study. Individual consents for intervention were obtained from all patients. The study database includes pre-operative demographic data, anatomical characteristics, procedural details, and follow-up outcomes, including post-operative clinical events and imaging results.

### 2.2. Patient Selection

Different descending thoracic aorta pathologies were treated, including type B aortic dissection, penetrating aortic ulcer (PAU), and thoracic aortic aneurysm (TAA). Based on their anatomy, age, and existing comorbidities, all patients were considered at high-risk for open repair. The treatment strategy was individually tailored by a multidisciplinary aortic team based on patient-specific factors.

The authors considered the Castor device in case of aortic lesions requiring TEVAR in Ishimaru proximal landing zone 1 or 2, with:

− proximal landing zone length ≥15 mm;
− proximal landing zone diameter <40 mm;
− distance of at least 5 mm between the target vessel for the branch and the ostium of the more proximal vessel;
− distance of at least 25 mm between the left vertebral artery origin and LSA ostium.

Pre-operative computed tomography angiography (CTA) was evaluated for all patients using dedicated software.

All patients who received the Castor stent-graft at the participating center during the study period were included in the analysis. Exclusion criteria were patients who were not suitable for endovascular aortic repair or were unable to give informed consent.

### 2.3. Stent-Graft Characteristics and Procedural Details

**Figure 1:**
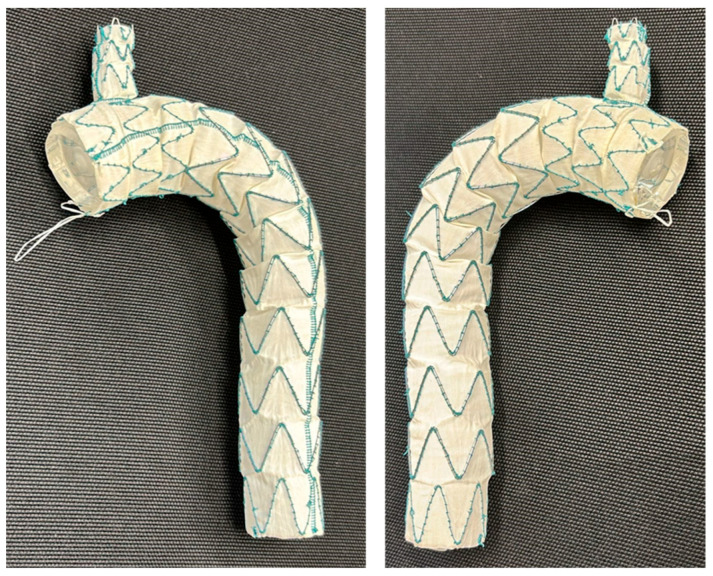
Castor single-branched thoracic aortic stent graft (Microport Medical, Shanghai, China).

The stent-grafts are constructed from a blend of nitinol and polyester, comprising a main body and a solitary branch (Figure 1).

The device’s delivery system is composed of an outer 24 Fr catheter, a soft inner sheet inside which the stent-graft is loaded, and a branch traction wire. Different configurations of this device are available according to some key parameters: the proximal and distal diameters and the length of the main body, the diameter and the length of the branch, and the distance from the ostium of the branch to the proximal end of the main body.

According to center preferences, the procedures were conducted either under general or local anesthesia and with both surgical and/or percutaneous access in a hybrid room setting. Systemic heparin (100 units/kg) was administered, and the activated clotting time was monitored (target > 300 s). The deployment of the endograft involves the main femoral access and a left brachial/axillary or left carotid access, depending on the target vessel of the branch. The cranial access allows for capturing the traction of the through-and-through floppy buddy wire preloaded into the branch. Once this is done, the delivery system is advanced over a stiff guidewire, positioned in zone 1 or 2, and rotated to align the branch with the greater curvature, ensuring that the traction wire does not get entangled around the delivery sheath. Then the outer sheath is retracted, and the stent-graft is cautiously advanced into the aortic arch while the branch is pulled via the traction wire into the target vessel. Once the graft reached the target position, the inner soft sheath was removed. Finally, the stent-graft and then the branch are completely deployed. Hemodynamic adjuncts during proximal deployment were performed according to the center’s preferences. Completion angiography was performed in the anteroposterior (AP) and right anterior oblique (RAO) projections for all patients. In cases involving PLZ 1, concomitant cervical debranching was performed before the TEVAR procedure using a Dacron graft (Gelweave, Terumo Aortic, Sunrise, FL, USA).

### 2.4. Postoperative Management

Following the procedure, the patients underwent single or double antiplatelet therapy (DAPT) according to center preference. When prescribed, DAPT was continued for 3 months and then switched to single antiplatelet therapy. Patients were observed at regular post-operative appointments. Follow-up with CTA was scheduled at 1 and 6 months postoperatively, followed by yearly imaging.

### 2.5. Endpoints and Definitions

Outcomes were assessed based on the current reporting [7]. The primary endpoints included technical success, the rate of intraoperative major adverse events (MAEs), and deployment accuracy. Technical success was defined as the correct placement of the endograft at the intended anatomical site, with no evidence of type I or III endoleaks, and with confirmed patency of both the graft and all involved supra-aortic trunks (SATs) on intraoperative angiography. Intraoperative MAEs were defined as a composite measure that included any intraoperative death, need to convert to open surgery, or occurrence of aortic rupture. Deployment accuracy was considered achieved when the graft was positioned within 5 mm of the planned proximal landing zone. Secondary endpoints were aortic-related mortality, all-cause mortality, aortic-related reinterventions, occurrence of endoleaks (ELs), neurological events, and endograft-related complications both at 30 days and throughout the follow-up period. Neurological complications included ischemic stroke and transient ischemic attack (TIA), defined as new neurological deficits lasting more or less than 24 h, respectively. Early events were classified as those occurring within the first 30 days post-procedure or during the index hospitalization.

### 2.6. Statistical Analysis

Statistical analysis was performed utilizing SPSS version 22.0 (SPSS Inc., Chicago, IL, USA). Continuous variables were presented as means accompanied by standard deviations (±SD), as well as medians with interquartile ranges (IQR), according to the data’s distribution. Furthermore, categorical variables were expressed in terms of frequency and percentage, allowing for a clear and concise representation of demographic and clinical characteristics.

## 3. Results

### 3.1. Study Population

Between January 2019 and April 2025, 51 patients with different thoracic aortic diseases who underwent TEVAR with the Castor stent-graft were included in the analysis. Baseline characteristics and type of pathologies are detailed in Table 1.

The mean age was 68.8 years ± 8 years. Most of the patients were male (75.5%), with a high prevalence of arterial hypertension (74.55%). The most common indication for use of the Castor device was type B aortic dissection (45.1%), followed by TAA (27.5%), PAU (19.6%), and IMH (5.8%). Preoperative measurements obtained through CTA showed a median PLZ length of 30.6 mm (IQR 26–34 mm).

### 3.2. Anatomical Details

The median proximal and distal landing zone diameter was 30.6 mm (interquartile range 26.7–33.8) and 32 mm (interquartile range 28.3–37.3), respectively. The median LSA diameter was 10 mm (interquartile range 9–11.7). Patients presented an arch type I in 33 (64.7%) cases, type II in 10 (19.6%) cases, and type III in 8 (15.7%) cases.

### 3.3. Procedural Details

**Figure 2:**
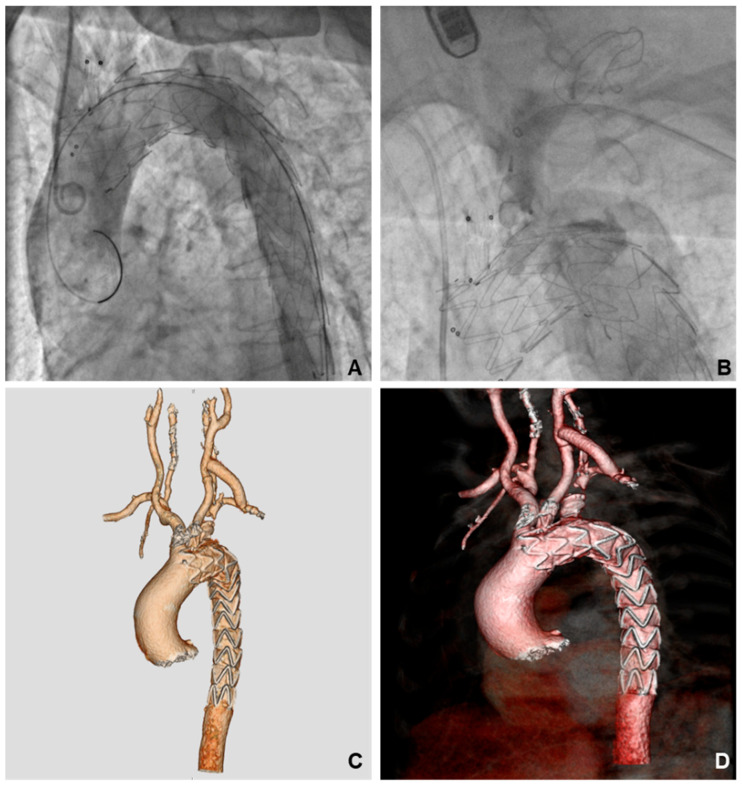
(**A**) Angiographic control after graft release in landing zone 1 with the branch in the left common carotid artery. (**B**) Angiography during plug deployment in left subclavian artery. (**C**,**D**) Post-operative three-dimensional volume rendering computed tomography.

Table 2 provides a comprehensive summary of procedural details and intraoperative results. Proximal deployment was zone 1 (Figure 2) in 4 cases (7.8%), and zone 2 in 47 cases (92.1%). SAT debranching was performed in 4 patients (7.8%).

The procedure was performed under general anesthesia in 42 cases (82.3%) and local anesthesia in 9 cases (17.6%). The overall technical success rate was 94.1% (n = 48), due to 3 cases (5.8%) of type Ia endoleak detected intraoperatively. No other endoleaks were detected.

The median fluoroscopy duration was 35 min (IQR 18–50); the median contrast volume used was 150 mL (IQR 80–270). No intraoperative MAEs, including stroke and deaths, occurred.

### 3.4. Early Outcomes

A total of two cerebrovascular events (3.9%) were reported, both classified as minor embolic strokes occurring during TEVAR procedures with proximal landing in zone 1. In both cases, neurological symptoms fully resolved without long-term deficits. No instances of spinal cord ischemia (SCI) were observed. Access-related complications occurred in two patients (4.1%) who underwent femoral artery cut-down; both experienced wound dehiscence associated with lymphatic leakage. No cases of in-hospital mortality or retrograde aortic dissection were documented. At the 1-month postoperative CTA, three cases (5.8%) of type Ia endoleaks were observed. One patient (2.0%) who underwent TEVAR with zone 2 proximal landing developed a type Ia endoleak, which was successfully treated four months later with a carotid-subclavian bypass and proximal relining in landing zone 1. The remaining two cases declined further intervention and were managed conservatively with ongoing surveillance, showing no sac enlargement over time. Early outcomes are detailed in Table 3. During a mean follow-up of 22.3 months (range: 2–58 months), no additional type Ia or type III endoleaks were observed. All SAT bypass, grafts, and branches were patent throughout the follow-up period. Three patients (5.8%) died during follow-up, with all deaths unrelated to aortic pathology.

## 4. Discussion

The advent of total endovascular options for the treatment of aortic arch and thoracic aortic pathologies has revolutionized the management of aortic diseases, providing a less invasive alternative to traditional open surgery [1]. The ability to perform aortic repairs without the need for sternotomy or the risks associated with open surgery has expanded treatment options for patients who were previously deemed ineligible because of advanced age, comorbidities, or anatomical challenges. Fenestrated and branched thoracic grafts have become viable solutions for patients with complex aortic pathology involving Ishimaru’s proximal landing zones 1–2 [8]. A recent meta-analysis on endovascular devices for partial and total repair of the aortic arch in 571 patients showed that the most used configuration is the branched TEVAR (54.3%), and that lesions requiring treatment in zone 1–2 represents 32.4% of TEVAR [8].

In the present Italian multicenter study, we reported the early outcomes of TEVAR using the Castor branched aortic stent graft in a real-world setting. Although this device was originally designed for the treatment of type B aortic dissection requiring proximal fixation in Ishimaru zones 2 and 3, its use has been increasingly extended in routine clinical practice due to several distinctive design advantages. In our experience, the Castor device was used in 7.8% of cases with proximal landing in zone 1, for aneurysmal disease in 27.5% of cases. This broader application reflects growing confidence in the device’s performance and its adaptability to different aortic anatomies beyond its initial indications.

It features a unibody design, in which the main aortic component and the outer branch are integrated. This configuration enables simultaneous deployment of both elements, which not only simplifies the implantation process but also reduces inter-component junctions and device migration risks. Moreover, the delivery system composed of an outer hard sheath, an inner soft sheath, and the sheath of the branch section can assure precise graft placement. The orientation and position of the branch are guided by a patented wire-controlled, pull-in design. The crucial point is a careful check that the guidewires are not twisted/wrapped around the delivery system before the graft release.

Currently, the Castor branching aortic stent graft system has demonstrated safe early outcomes [6,9]. Ren et al. and Zolnierczuk et al. reported 98.1% and 95.2% rate of technical success in their multicenter studies with this device, respectively [6,9]. In our experience, the technical success rate was 94.1%, due to 3 cases of type Ia endoleak detected intraoperatively. Type Ia endoleaks represent a significant concern during TEVAR, with a reported incidence of 21.3% and an increased risk in case of proximal landing zone [10,11]. It is reported that branched endografts present a lower rate of type Ia endoleaks than fenestrated endografts (2.6% vs. 9.8%; *p* = 0.034) [8]. In our series, the total rate of type Ia endoleaks was 5.8%. The Castor device presents a 5–30 mm length from the branch to the main body proximal end. However, the design ensures that proximal sealing is achieved not only by the main stent graft but also by the branch-bearing segment itself. In our study, the type Ia endoleaks occurred in 1 case in zone 2 with 15 mm proximal length requiring proximal relining, and in 2 cases of thoracic aortic aneurysm with proximal landing zone 1 with 39 mm diameter. Proximal relining in zone 1 was performed with a carotid-carotid subclavian bypass and concomitant TEVAR without any complications.

In the present study, the stroke was reported in two patients (3.9%). Early studies with the Castor device have reported low rates of stroke, ranging between 0.6% and 4.8% [6,9,12,13,14,15]. A recent metanalysis on 22,244 patients showed that the overall risk of clinically evident stroke during TEVAR is 2.7% for zones ≥ 3, 6.6% for zone 2, and 7.7% for zone 1 [16]. The pooled rate of strokes reported by Spath et al. was 6.2% with no differences between branched and fenestrated endografts (6.8% vs. 5.3; *p* = 0.5) [8]. Notably, all our observed strokes (3.9%) occurred in zone 1 cases where the side branch was deployed to the left common carotid artery instead of the LSA, suggesting a potential correlation between target vessel selection and embolic risk. The preloaded branch stent-graft system in zone 1 may have introduced a higher embolic burden due to instrumentation across the aortic arch and direct manipulation within the carotid vessel. Despite these events, full neurological recovery was achieved in both cases. However, these events underscore the need for careful patient selection and potentially adjunctive protective strategies when planning zone 1 interventions with carotid branch incorporation.

One of the key advantages of this branched device is the ability to preserve perfusion to LSA without the need for surgical debranching, thereby minimizing operative trauma and reducing the risks associated with hybrid procedures. Carotid-subclavian bypass has generally been considered a safe and effective technique, with a reported stroke incidence of 4.3% and a primary patency rate of 98.1% at 15-month follow-up [17,18]. However, the procedure is not without risks; published data indicate postoperative complication rates of 11.4% for local bleeding, 10.4% for reintervention, and 9.5% for peripheral neurological injury, highlighting the potential morbidity associated with surgical debranching [17,18,19]. A study on 837 patients comparing carotid-subclavian bypass or transposition vs. total endovascular techniques for LSA revascularization during TEVAR showed no differences in terms of stroke and endoleaks [20].

The large delivery profile (24 Fr) of the Castor device necessitates adequate iliofemoral access and may increase the risk of access-related complications. In our experience, no access complications were observed because of the accurate patient selection. Additionally, the complexity of the deployment process, including the need to manage the traction wire and its interaction with the stiff guidewires, requires a steep learning curve.

## 5. Limitations

The study presents some limitations that warrant careful consideration when interpreting its findings. The relatively small sample size raises concerns about the statistical power and generalizability of the results. Furthermore, the follow-up duration restricts the ability to assess long-term outcomes and potential complications associated with the intervention. A consistent and longer-term follow-up is essential to evaluate the risk of late complications. Future studies with extended surveillance and larger cohorts will be necessary to confirm these early and mid-term results.

## 6. Conclusions

Our early results suggest that TEVAR paired with the Castor device may provide a viable treatment option for aortic arch and thoracic aortic diseases, particularly in PLZ 2. Further studies with long-term data are necessary to assess the benefits and durability of this endograft.

## Figures and Tables

**Table 1:** Baseline features and treatment indications.

	n = 51	%
***Baseline details***		
Male	37	75.5
Mean age ± SD	68.8	±8.0
Smoking	10	19.6
Hypertension	38	74.5
Diabetes mellitus	5	9.8
Dyslipidemia	26	51
CKD		
Stage 1–3a (eGFR > 44)	43	84.3
Stage 3b–4 (eGFR 15–44)	6	11.7
Stage 5 (eGFR < 15)	2	3.9
COPD	2	3.9
Cerebral vessels disease	2	3.9
AMI	5	9.8
Previous CABG	0	0
Previous PCI	5	9.8
ASA score		
ASA II	18	35.3
ASA III	7	13.7
ASA IV	26	50.9
Antiplatelet Therapy	34	66.7
Anticoagulation Therapy	9	17.6
***Aortic Pathology***		
Type B Aortic Dissection	23	45.1
Thoracic aortic aneurysm	14	27.5
Penetrating aortic ulcer (PAU)	10	19.6
Intramural Hematoma	3	5.8
Left subclavian artery isolated aneurysm	1	1.9

SD, standard deviation; COPD, chronic obstructive pulmonary disease; CKD, chronic kidney disease; eGFR, estimated Glomerular Filtration Rate calculated CKD-EPI Creatinine Equation (2009) and measured in mL/min/1.73 m^2^; AMI, acute myocardial infarction; CABG, coronary aortic bypass graft; PCI, percutaneous coronary intervention; ASA score, American Society of Anesthesiologists score.

**Table 2:** Procedural Characteristics and Intraoperative Outcomes.

	n = 51	%
***Procedural Characteristics***		
Ishimaru proximal landing zone		
Zone 1	4	7.8
Zone 2	47	92.1
Supra-aortic debranching		
Carotid-subclavian bypass	4	7.8
Left Subclavian Artery Plug	4	7.8
Anesthesia		
General	42	82.3
Local	9	17.6
Femoral Access Approach		
Percutaneous	41	80.4
Cut-down	10	19.6
Systolic pressure during proximal graft release		
100–80 mmHg	8	15.7
79–60 mmHg	40	78.4
<60 mmHg	3	5.8
Hemodynamic adjuncts during proximal deployment		
None	10	19.6
Pharmacological Hypotension	29	56.9
Rapid Cardiac Pacing	12	23.5
Operating time, min	110	Range 40–180
***Intraoperative Results***		
Technical success	48	94.1
Access Failure	0	0
Deployment Failure	0	0
Intraoperative Endoleak		
Type Ia	3	5. 8
Type Ib	0	0
Type II	0	0
Type III	0	0
Intraoperative adjunctive graft-related procedures	0	0
Major Adverse Events	0	0
Intraoperative stroke	2	0
Intraoperative death	0	0

**Table 3:** Early results.

Early Results	n = 51	%
Cerebrovascular complications	2	3.9
Major Stroke	0	0
Minor Stroke	2	3.9
Spinal Cord Ischemia (SCI)	0	0
AMI	0	0
Acute renal failure	0	0
ARI	0	0
Access related complications	0	0
Endoleak		
Type Ia	3	5.8
Type Ib	0	0
Type II	0	0
Type III	0	0
Retrograde dissection	0	0
Early reintervention	0	0
In-hospital mortality	0	0
30-day mortality	0	0
Mean hospitalization time, days (SD)	3	±2
Mean ICU stay, days (SD)	0	±1

AMI, Acute Myocardial Infarction; ARI, Acute Respiratory Infection; SD, standard deviation; ICU, Intensive Care Unit.

## Data Availability

The data presented in this study are available on request from the corresponding author due to privacy restrictions.
